# Supplementary material for: The Influence of Calcium on the Growth, Morphology and Gene Regulation in Gemmatimonas phototrophica
Source: Microorganisms. 2022 Dec 22;11(1):27. doi: 10.3390/microorganisms11010027 (PMC9862903; doi:10.3390/microorganisms11010027)
Supplement: Supplementary file 1 [file microorganisms-11-00027-s001.zip › SI.pdf]

Supplementary Material

The influence of calcium on the growth, morphology and gene regulation of *Gemmatimonas phototrophica*

Sahana Shivaramu[1], Jürgen Tomasch[1], Karel Kopejtka[1], Nupur[1], Mohit Kumar Saini[1], Syed Nadeem Hussain Bokhari[2], Hendrik Küpper[2,3], Michal Koblížek[1]*

[1] Laboratory of Anoxygenic Phototrophs, Institute of Microbiology of the Czech Acad Sci, 37 981 Třeboň, Czech Republic

[2] Czech Academy of Sciences, Biology Centre, Institute of Plant Molecular Biology, Department of Plant Biophysics and Biochemistry, Branišovská 1760/31, 37005 České Budějovice, Czech Republic

[3] University of South Bohemia, Faculty of Science, Department of Experimental Plant Biology, Branišovská 1760/31a, 370 05 České Budějovice, Czech Republic

**Author for correspondence: koblizek@alga.cz*

This file contains:

Supplementary Figure S1.

Supplementary Datasets S1 and S2 are included as a separated Excel files.

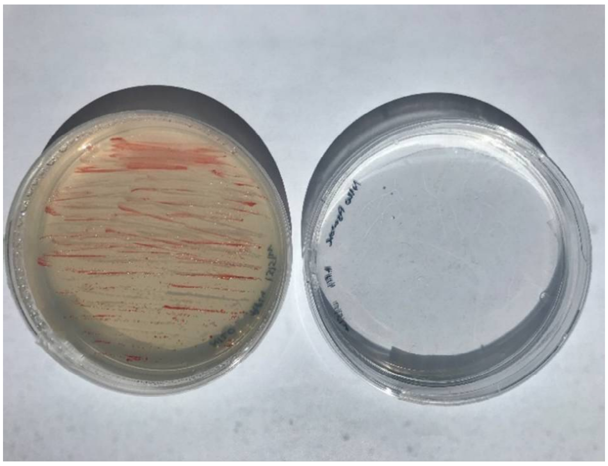

Supplementary Figure S1. Plates showing pure cultures of *G. phototrophica* growing on a medium solidified by agar (left) but not growing on a medium solidified by agarose (right).
